# Experimental and Theoretical Investigations of the Fragmentation of Ethylenediamine Induced by Low-Energy (<10 eV) Electrons

**DOI:** 10.3390/molecules29010191

**Published:** 2023-12-28

**Authors:** Hassan Abdoul-Carime, Elena Lys, Jeanne Gipouloux, Franck Rabilloud

**Affiliations:** 1 Université Claude Bernard Lyon 1, Institut de Physique des 2 Infinis, CNRS/IN2P3, UMR5822, F-69003 Lyon, France; 2 Université Claude Bernard Lyon 1, Institut Lumière Matière, CNRS/INP, UMR5306, F-69622 Villeurbanne, Francefranck.rabilloud@univ-lyon1.fr (F.R.)

**Keywords:** ethylene diamine, low-energy (<10 eV) electrons, dissociative electron attachment, ab initio calculations, anion and neutral mass spectrometry measurements

## Abstract

Ethylenediamine is industrially used as an intermediate for the fabrication of many products. The development of new methodologies for synthesis compatible with the environment and sustainability, such as cold plasma processes, implicates reactions induced by nonthermal electrons. In this contribution, we study the interaction of low-energy (<10 eV) electrons with ethylenediamine. We show that electrons induce the fragmentation of the molecule into various anion fragments and associated neutral counterparts via dissociative electron attachment. The fragmentation mechanisms and energetics are discussed in the frame of DFT calculations. The fragmentation processes are quantified by the estimation of the cross sections and the branching ratios for competitive accessible dissociation routes.

## 1. Introduction

Ethylenediamine (EDA), C_2_H_4_(NH_2_)_2_, is widely used in industry as a building block for chemical synthesis to produce detergents, chelates, textile auxiliaries, agrochemicals, and polyamides [1], and the market demand for this compound is continuously growing [2]. Recent decades have seen the development of research for promoting EDA in sustainable chemistry [3,4,5,6], such as the fabrication of nanosheets for the catalysis of H_2_ assisted by ethylenediamine [5] or the “green” production of polymers from γ-grafting methods of chitosan and EDA [6]. In irradiation techniques, energetic particles create various species (ions, electrons, and neutral radicals) that may further react, generating different byproducts. Obtaining the specific synthesized compound often requires steps of purification. In contrast, the development of selective synthesis via targeting a specific reactive site [7], can reduce the impact of substance overuse for more sustainability.

Light-induced site-specific chemical reactions are well established [8]. For instance, the UV-activated polymerization has already attracted industrial sectors for various applications [9,10]. Chemistry can also be triggered by electrons at low energies (<20 eV) [11]. Such a reaction can be particularly selective at sub-ionization energies [12,13], as has been shown, for instance, by the selective synthesis of ethylene from the irradiation of dimethyl sulfide films at a specific electron energy [13]. These slow electrons are produced by a traditional electron gun [11,12,13], scanning tunneling microscope (STM) [14], in plasma [15], or via surface plasmon resonance (SPR). For the latter, the surface reaction [16] arises from the transfer of hot <5 eV electrons into the surface target molecules [17] induced by the light-excited metal surface in ambient conditions. At these energies, the reactivity activated by electrons is controlled by energy resonant excitation or resonant electron capture by the molecule to form a transitory anion that can further decay into the fragmentation of specific molecular bonds (dissociative electron attachment, DEA) [18,19]. The fragmentation processes induced by low-energy electrons are to some degree similar to photons, but in general with much higher cross sections. Therefore, a comprehensive understanding of the physics and physical chemistry of the interaction of sub-ionization electrons with molecules in isolation (i.e., in gas phase conditions) is desirable prior to any further perspective studies and potential scalable applications [20].

Here, we show that the collision of low energy with ethylenediamine resonantly generates various negative species, i.e., NH^−^, CN^−^, and (EDA-H)^−^ and the metastable precursor EDA^−^ anions. The production of charged fragments is accompanied by one or more neutral counterparts that are identified experimentally. The resonant states and the energetics of the molecular fragmentation are calculated by ab initio calculations. The molecular fragmentation processes are quantified by the measurement cross sections and the branching ratios for observed competitive fragmentation channels.

## 2. Results and Discussion

**Figure 1:**
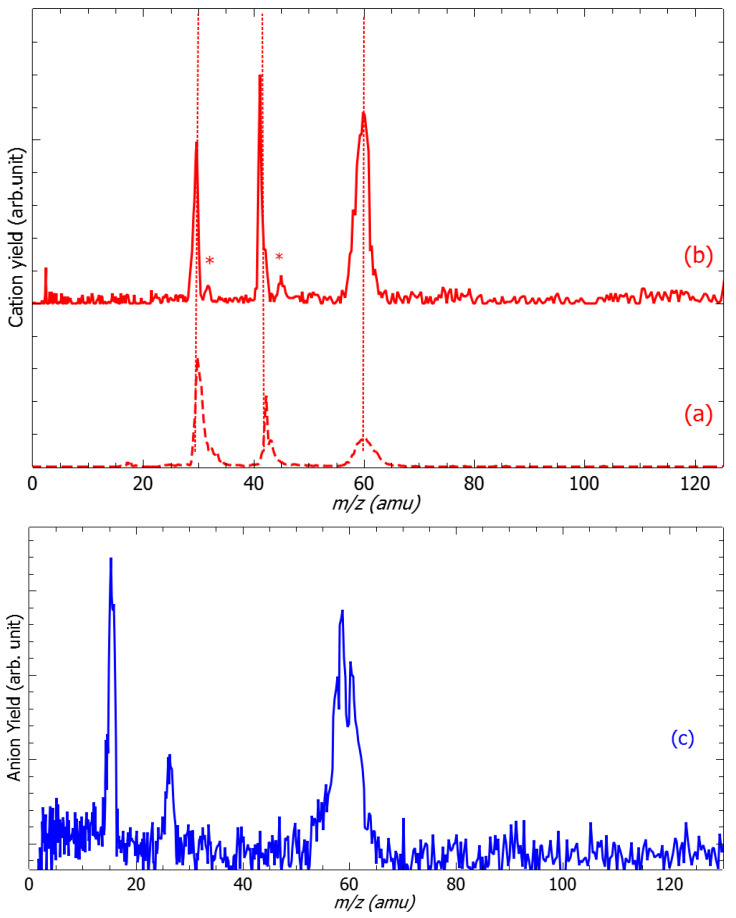
Cation (red) and anion (blue) mass spectra. (**a**) The cation mass spectrum (red dashed line) recorded at the electron energy of 16 eV from EG2 presents fragments agreeing with the NIST databank report [21], (**b**) (solid red line) recorded at the electron accelerating voltage from EG2 of 9.0 eV, EG1 set to 3.2 V exhibits additional features (red stars), and (**c**) the anion mass spectrum recorded at the electron accelerating voltage of EG1 of 3.2 V, EG2 set to “off” shows four peaks at *m*/*z* 16, 26, 59, and 69, respectively. The dotted lines are a visual guide.

Figure 1a (red dashed line) presents the cation mass spectrum recorded from the impact of 16 eV electrons produced by EG2 with EDA targets, while EG1 is set to “off” mode. It exhibits only peaks observed at the *m*/*z* of 30, 43, and 59 (shoulder) and 60 amu in agreement with those reported by the NIST databank [21]. This measurement clearly shows that the results presented in this contribution are obtained from the collision of low-energy electrons with monomers of ethylenediamine without any traces of contaminants. Figure 1c (blue solid line) exhibits the anion mass spectrum recorded at the colliding electron accelerating voltage of 3.2 V (EG1). The species at *m*/*z* of 15, 59 and 60 are attributed unambiguously to the NH^−^, (EDA-H)^−^ and EDA^−^ anions. In contrast, the *m*/*z* 26 can be assessed by stoichiometry to either the CN^−^ or the C_2_H_2_^−^ anion fragment. At low energies, the fragmentation of molecules induced by electrons arises via DEA [18,19]. In brief, the electron is captured by the target molecule to form a transitory negative ion (TNI). The TNI, which will be discussed below, may autodetach the excess electron, leaving the precursor in an excited state (that may further or not dissociate) or fragment producing a negative-ion fragment and one or more neutral counterparts. It is this latter process that is observed in this experiment. The production of (EDA-H)^−^ anion is accompanied by the radical neutral hydrogen, while the processes for the NH^−^ and *m*/*z* 26 negative species are more complex. To probe the associated neutral species associated with these anion fragments, the EDA targets are irradiated by pulsed counter-propagative electron beams from EG1 and EG2. The mass spectrum shown by the solid red line is recorded at the ionizing energy of 9.0 eV (EG2), while the collision electrons from EG1 accelerate at 3.2 V (i.e., 2.8 eV, see below). We observe additional features (labeled by asterisks) associated with *m*/*z* 31 and 45 amu fragments. The 45 amu ionized neutral species (C_2_NH_7_) may be associated with the corresponding NH^−^ anion and the 31 amu ionized neutral fragments (CNH_5_) with the CN^−^ anion. The ionization potential of C_2_NH_7_ and CNH_5_ are measured to be 9 eV [21] and 8.2 eV [21], respectively, allowing these neutral partners to be ionized by EG2 in the measurement conditions (Figure 1b).

**Figure 2:**
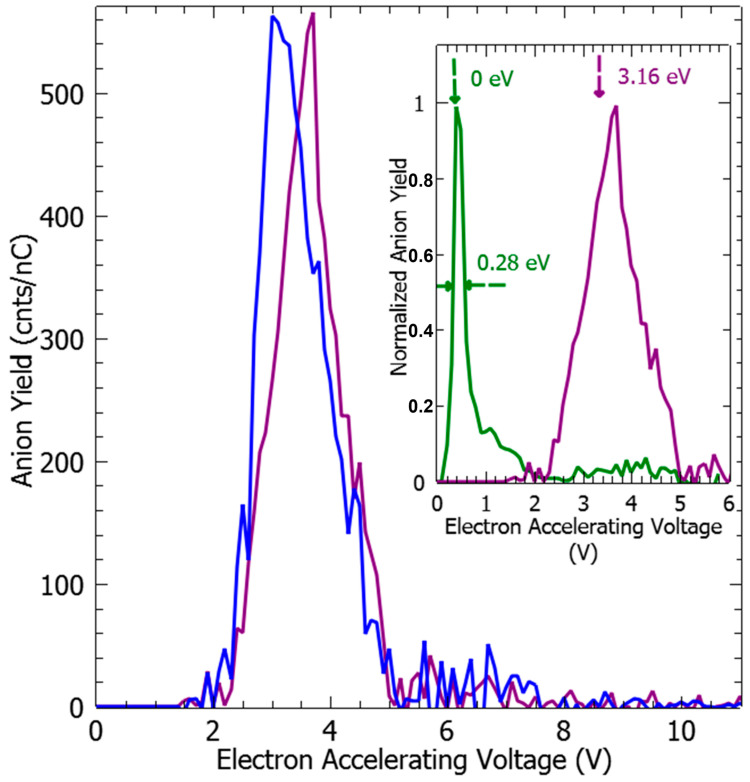
EDA^−^ (blue) and (EDA-H)^−^ (purple) anions yield as the function of the incident electron accelerating voltage. In the insert, the anion yield functions are obtained from electron impact on pure CCl_4_ and EDA vapors: the yield for Cl^−^ (green) and the (EDA-H)^−^ fragments are normalized to the respective maximum value. The full-width half-max in the Cl^−^ anion yield provides the energy resolution of the electron beam and the electron energy reference, i.e., 0 eV, associated with the maximum yield (see text).

**Figure 3:**
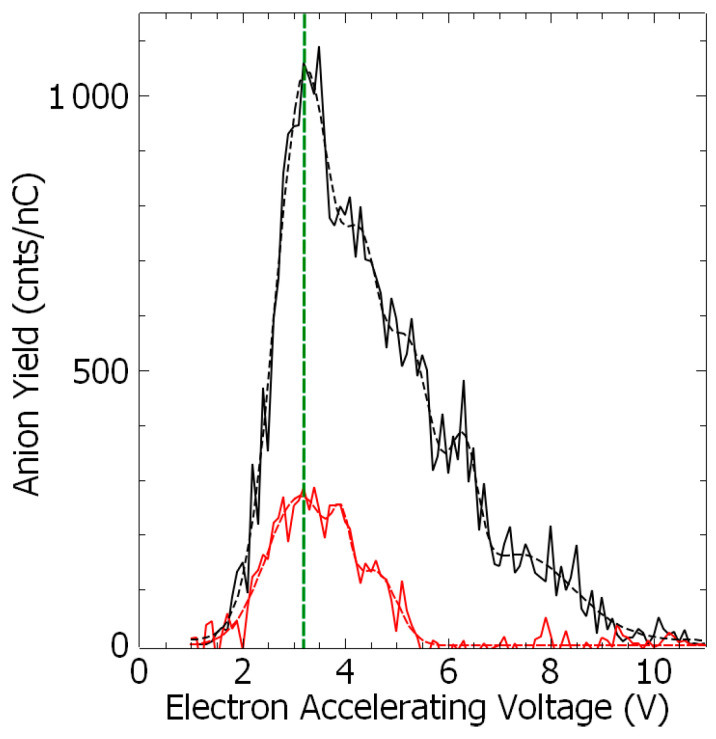
Yield of the anion fragment (NH^−^, black, and CN^−^, red) as the function of the incident electron accelerating voltage. The dashed lines are a visual guide. The green dashed line corresponds to the peak position of 3.1 eV (see Table 2).

One of the prerequisites for the anion to be formed concerns the energetics of fragmentation. Table 1 presents the energy threshold calculated at 0 K and the free Gibbs energy evaluated at 300 K for the observed anions. The production of (EDA-H)^−^ anion arises from a simple N–H or C–H bond cleavage (Figure 2). The calculations show that the bond rupture at the nitrogen sites (i.e., Table 1 (a) and (b)) is generally energetically favored by ~0.6 eV, which is in relatively good agreement with the experimental observation of the threshold appearance of the anion. At higher energies, the loss of the hydrogen atom may arise from any site. The formation of the NH^−^ anion may arise from two possible routes, i.e., (e) et (f) (Table 1). Below 6 eV, it is very likely that the NH^−^ anion (Figure 3, black) is formed along with its CH_3_CH_2_NH_2_ neutral counterpart. It is noteworthy that the reaction requires a swing over of the hydrogen atom from the nitrogen site to the –CH_2_ site, and is thus a potential barrier to overcome that is not included in the estimated energetics (Table 1 (f)). However, the fact that the ionized C_2_NH_7_ species is observed (Figure 1b, labeled *) supports the reaction (f) (Table 1). Above 6 eV, both reactions (e) and (f), are a priori accessible. The CN^−^ anion is the last detected species. From its yield function (Figure 3, red), the reactions (h) and (i) described in Table 1 are accessible. Indeed, the neutral species associated with reaction (i), i.e., CNH_5_, is detected in the cation mass spectra at *m*/*z* 31 (Figure 1b, labeled *), while that associated with (h), i.e., CNH_4_ (*m*/*z* 30) must be overlapped by the cracking pattern of the ionization spectrum of ethylenediamine [21]. Alternatively, regarding the production of the CN^−^ anion, that of the C_2_H_2_^−^ anion via the reaction (j) (Table 1) is also energetically accessible. Nonetheless, this reaction must involve the swing over of an H atom from each NH_2_ site (i.e., additional potential barrier for the reaction), leading to a closed-shell (H–C≡C–H)^−^ anion, a radical (H–C=C–H)^−^ anion, or a multi-radical (H–C–C–H)^−^ anion and two NH_3_ molecules. It is noted that the trans-bent radical acetylene (H–C≡C–H)^−^ anion has been observed in low-temperature radiolysis of the alkane matrix experiments [22,23]. In the gas phase, the acetylene radical anion is unstable towards the electron loss in comparison to the vinylidene (H_2_C=C)^−^ anion [24]. Forming this latter product in the present dissociative electron attachment experiments would require a sufficiently long time for atomic and bonding rearrangements. Similarly, further possible calculated fragmentation routes are a priori accessible energetically (Table S1), but the suggested anion fragments are not observed in the present work for two possible reasons: (1) the signal is below the detection limit (i.e., low fragmentation cross sections) or (2) the dissociation time is larger than the electron autodetachment time [18,19], as discussed below.

**Figure 4:**
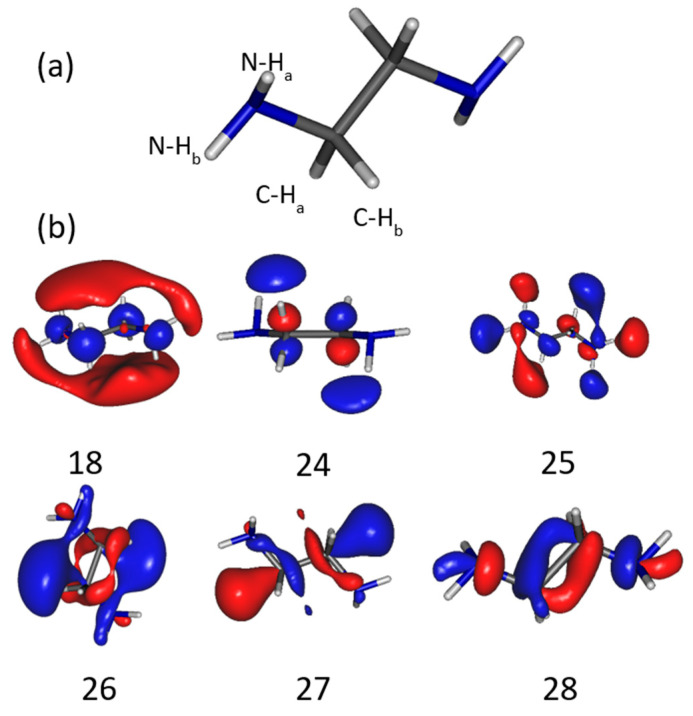
(**a**) Structure of EDA showing the atoms labeled H_a_ and H_b_ involved in the fragmentations mentioned in Table 2. (**b**) Molecular orbitals of the EDA^−^ anion. Orbital 18 is the HOMO, while the others are unoccupied orbitals in the ground state.

As the energetics of the fragmentation are discussed, the second prerequisite for the anion production relies on the specific electron–molecule collision process at energies below the ionization of the molecule, i.e., <9–10 eV. At a given accelerating voltage value of the collision electron (EAV from EG1), the anion yield is obtained by integrating the peak of the specific *m*/*z* value of the mass spectrum and by subtracting the respective background noise (Figure 1c) and normalized to the transmitted electron current. Figure 2 and Figure 3 show the anion yield as the function of the electron accelerating voltage, EAV. In these measurements, EG2 is set to “off” mode. In the inset of Figure 2, the yield function of the (EDA-H)^−^ and Cl^−^ anions are normalized to the maximum of their respective yield. As the Cl^−^ anion is well known to be produced at ~0 eV incident electron energy [25], the electron energy scale is obtained by shifting the accelerating voltage EAV scale by 0.4 eV. Thus, the peak in the (EDA-H)^−^ yield function (Figure 2, solid purple line) is observed with an electron attachment energy of 3.16 eV. The observed peak positions in correlation with the calculated resonance states are listed in Table 2. As seen in Figure 2 and Figure 3, the anion yield functions exhibit structures indicative of resonant processes initiated by the electron attachment followed by the dissociation of the transitory formed EDA^#−^ anion. Dissociative electron attachment is controlled by three processes: (1) the energetics of the fragmentation, (2) the capture of the excess electron in a molecular orbital (MO), and (3) the survival probability, i.e., the dissociation time vs. the electron autodetachment time; (1) is already discussed above for different fragments (Table 1). The capture of the extra electron may arise from the shape resonance (i.e., accommodation into a usually unoccupied MO), core excited electron (i.e., excitation of a valence electron into an MO, concomitantly trapping of the extra electron by the positive core) or multipole-bound initiated vibrational Feshbach resonance [18,19]. The main calculated MOs are provided in Table 2 and shown in Figure 4. In the electronic ground state, the extra electron is stabilized in the orbital 18 (MO 18) via a long-range interaction between the extra electron and the electric quadrupole moment of the molecule. Indeed, EDA does not present a dipole moment but a high quadrupole moment (c.a., XX = −23.9327, YY = −26.1189 ZZ= −28.0714, in D.Å). The anion is likely associated with quadrupole bound anions that cannot be observed in this experiment but can be in Rydberg electron transfer-type experiments [26]. The attachment of the electron in the first excited state calculated at 2.81 eV is mainly associated with the MO 24, which is of π* character, and the lifetime of this state is expected to be long enough for the anion to be detected in the experiment. The calculated metastable EDA^−^ anion agrees with the experimental observation (Figure 2, blue). The electron attachment calculated at 3.01 eV is associated with the MO 25, which presents a σ* character along each bond. It may lead to the production of (EDA-H)^−^, CN^−^, and NH^−^, measured at 3.16, 3.07, and 2.94 eV, respectively. The main peak near 4 eV is due to the fragmentation in CN^−^ (4.23 eV) and NH^−^ (3.93 eV), and may be associated with the attachment calculated at 4.36 and 4.46 eV, particularly the latter, which presents a σ* character (MO 27) that is favorable to C–C bond cleavage. At higher energy, the fragment NH^−^ is measured at 5.92 eV, which can correspond to the attachment calculated at 5.75 eV that presents a σ* character along the C–N bond (MO 28). In the calculation, several core-excited states are obtained above 5 eV. It is not mentioned here that the calculated resonance energies may differ slightly from the observed peak positions, since the dissociative electron attachment cross section results from the convolution of the attachment cross section and the survival probability, and both are energy-dependent.

As shown in Figure 2 and Figure 3 and Table 2, the transitory EDA^#−^ anion is formed at ~3.1 eV fragments into different channels producing the (EDA-H)^−^, the *m*/*z* 26, and the NH^−^ anion, for which the branching ratios are estimated to be 29%, 15% and 56%, respectively. These ratios reflect to some degree the dissociation time for which the dissociation time in the channel leading to the NH^−^ anion must be shorter than that to the *m*/*z* 26 negative species. Indeed, the ion DEA cross section, σ_i_, can be expressed ^Ilenb^ as σ_i_ = σ_o_ × P_s_^i^, where σ_o_ is the electron capture cross section into the transitory EDA^#−^ anion state and the survival probability, P_s_^i^ = exp(−τ_d_^i^/T_a_), τ_d_^i^ representing the dissociation time and T_a_ the electron-autodetachment time. Thus, T_a_.ln(σ_i_/σ_i_^ref^) is directly related to the change in the dissociation time, Δτ(ι.ε., τ_s_^i^ – τ_d_^i,ref^). Thus, taking the production of the NH^−^ anion fragment as the reference, we can estimate the change in dissociation time, using the time unit T_a_, to be −0.658 and 1.317, respectively, for (BZN–H)^−^ and *m*/*z* 26. At the resonance energy of ~4 eV, the branching ratios for the *m*/*z* 26 and the NH^−^ anion are estimated to be 21% and 79%, respectively.

Finally, the dissociation reaction can be quantified by the relative fragmentation cross section to that of the Cl^−^ anion from DEA to CCl_4_ [27] via N_ion_ # N_e_.N_mol_.L.σ_ion_ (with N_ion_ and N_e_ the number of the measured ions and colliding electrons, the density of the target molecules N_mol_, L the collision length and the fragmentation cross section σ_ion_). As the admixture of EDA:CCl_4_ gas (1:1.2) is injected for collision experiences the same L and N_e_, the relative fragmentation cross section at a given collision electron energy, E, can be estimated by: σ_ion_/σ_Cl_^−^ = (N_ion_/N_Cl_^−^).(P_CCl4_/P_EDA_). Using the established absolute cross section of 9.8 · 10^−15^ cm^2^ for the Cl^−^ anion production from the near-0 eV DEA experiment [25], the cross section for the production of (EDA-H)^−^ anion at 3.16 eV is estimated to be about 1.4 · 10^−15^ cm^2^. From Table 2 and Figure 2 and Figure 3, the cross section for all negative fragments and at the investigated energy range can be accessible. It is to be noted that the co-presence of the calibration gas (CCl_4_) does not lead to further reaction observable near 0 eV. For instance, in the low-energy electron impact of thymine, T, the presence of the carbon tetrachloride molecule in the thymine gas beam exhibits an additional dehydrogenated (T-H)^−^ anion near 0 eV [28], most likely resulting from the reaction of the produced Cl^−^ anion with T.

## 3. Methods

### 3.1. Experimental Method

The collision of low-energy (<10 eV) electrons with ethylenediamine targets is experimentally studied with a crossed beam setup, maintained at a base pressure of 5 × 10^−9^ mbar. The apparatus, thoroughly described elsewhere [27], comprises counter-propagating electron beams produced by two separated electron guns (EG1 and EG2), and a dual (+/−) time-of-flight mass spectrometer (TOFMS) positioned orthogonally to the electron beams. An effusive beam of EDA (99% purity Alfa Aesar, vapor pressure of 16 mbar) of 2 × 10^−6^ mbar is injected perpendicularly to both the electron beams and the TOFMS. EG1 is used for studying the electron–molecule collision process. It is equipped with a trochoidal monochromator, based on a dispersive ExB field) (an applied magnetic field of 80 G is aligned to the electron beams) providing an electron energy of resolution of 0.4 eV for a current of 8 nA. EG2, only used for ionizing the neutral species, does not require any specific electron energy resolution. The two TOF-MS are mounted face to face with a common extraction area. The positively and/or negatively charged ions formed are expelled oppositely from this area by a (−450 V, 600 ns) pulse at a rate of 5 kHz. They reach their respective acceleration areas (biased with +1450 V and −2000 V for the negative and positive ions, respectively) before traveling a free field area for the time dispersion. The ions are detected by a pair of microchannel plates mounted in chevon and the signal is preamplified before entering the acquisition (constant fraction discriminator, time-to-digital converter) and the data storage chain. The recorded arrival time of ions in reference to the extraction pulse represents the time of flight. The histogram built from the time of flight provides the mass spectrum, after time of flight-to-*m*/*z* conversion [27].

### 3.2. Theoretical Method

Calculations have been performed in the framework of the density-functional theory (DFT) and the time-dependent DFT (TDDFT) using the Gaussian16 suite of programs [29]. The exchange and correlation potential is that of the range-separated hybrid density functional ωB97x [30]. Enthalpies at 0 K and the Gibbs free energies of the fragmentation at 298 K are calculated using the diffuse basis set aug-cc-pvtz [31]. The resonance electron attachment energies are calculated with a multi-basis-set TDDFT method [32]. The vertical electron affinity of EDA is calculated at 0.02 eV using the basis set aug-cc-pvtz completed by diffuse s and p functions, while the anionic excitation energies are calculated using TDDFT and the cc-pvtz basis set. The use of the relatively small basis set cc-pvtz (without any diffuse function) is well suited to describe valence-type excitations while preventing the occurrence of intruder discretized continuum states. The method was found to furnish similar results to those obtained plotting the graph stabilization with the basis set aug-cc-pvtz [32]. Pre- and postprocessing operations are performed by using the graphical interface Gabedit (https://gabedit.sourceforge.net/, accessed on 25 December 2023) [33].

## 4. Conclusions

In the present study, we show that the collision of low-energy (<10 eV) electrons with ethylenediamine produces the metastable EDA^−^ anion, and also the fragmentation of the transitory precursor anion into the (EDA-H)^−^ NH^−^ and CN^−^ anions and their associated neutral counterparts, which can only be observed using the present methodology [27]. The transitory negative-ion states are calculated and the fragmentation thresholds are rationalized by the calculated energetics. The gain information from this investigation may potentially apply to various fields. For instance, the formation of CN^−^ anion is accompanied by CH_3_NH_2_ (methyl amine), and this latter species is important for the interstellar chemistry [34]. The knowledge of the fragmentation scheme and the associated cross section may be introduced into code simulating the evolution of interstellar media (ices, etc.) under cosmic ray irradiation.

## Figures and Tables

**Table 1:** Energy (in eV) for the production of anion and corresponding neutral fragment(s), calculated at ωB97x/aug-cc-pvtz level of theory at 0 K and Gibbs free energy at 298 K. The labels H_a_ and H_b_ are shown in Figure 4a.

Reaction	Fragmentation Energy (0 K)	Gibbs Free Energy (298 K)
EDA → (EDA-H)^−^ + H		
(a) N–H_a_	4.68	3.31
(b) N–H_b_	4.05	3.30
(c) C–H_a_	5.26	3.96
(d) C–H_b_	4.63	3.89
(e) EDA → NH^−^ + H + CH_2_CH_2_NH_2_	7.91	6.50
(f) EDA → NH^−^ + CH_3_CH_2_NH_2_	3.24	2.55
(g) EDA → CN^−^ + 2H_2_ + CH_2_ +NH_2_	8.61	5.94
(h) EDA → CN^−^ + 2H_2_ + CH_2_NH_2_	3.32	1.42
(i) EDA → CN^−^ + H_2_ + H + CH_3_NH_2_	3.74	1.93
(j) EDA → C_2_H_2_^−^ + 2 NH_3_	2.95	1.83

**Table 2:** List of the recorded anion fragments, peak positions (in eV) from Figure 2 and Figure 3 and the correlated calculated resonance states of the transitory EDA^#−^ anion. The branching ratio of the anion fragment production at a given energy is provided in parentheses.

Anion Fragment	Peak Position (±0.15 eV)	Calculated EDA^#−^ Resonance State (eV)	EDA^#−^ Molecular Orbital (Figure 4)
EDA^−^	2.72	2.81	24
(EDA-H)^−^	3.16 (29%)	3.01	25
CN^−^	3.07 (15%)	3.01	25
	4.23 (21%)	4.36, 4.46	26, 27
NH^−^	2.94 (56%)	3.01	25
	3.93 (79%)	4.36, 4.46	26, 27
	4.80		
	5.92	5.75	28
	6.99		

## Data Availability

Data are contained within the article and supplementary materials.

## Supplementary Materials

The following supporting information can be downloaded at https://www.mdpi.com/article/10.3390/molecules29010191/s1. Figure S1: NH^−^ (a) and CN^−^ (b) anion yield. The dashed lines are guide-to-the-eye. Table S1: Energy (in eV) for the production of anion and corresponding neutral fragment(s), calculated at ωB97x/aug-cc-pvtz level of theory at 0 K and Gibbs free energy at 298 K. The labels H(a), H(b) are shown in Figure 4a.

## References

[1] Eller K., Henkes E., Rossbacher R., Höke H. (2005). Amines aliphatic. Ullmann’s Encyclopedia of Industrial Chemistry.

[2] The Global Ethylenediamine (EDA) market size increase from 520 to 627 Kilo Tons from 2017 to 2022 and It Is Expected to Reach 831 Kilo Tons in 2027. https://www.hdinresearch.com/news/257.

[3] Fang L.J., Li Y.H., Liu P.F., Wang D.P., Zeng H.D., Wang X.L., Yang H.G. (2017). Facile fabrication of large aspect ratio g-C3N4 nanosheets for enhanced photocatalytic hydrogen evolution. ACS Sustain. Chem. Eng..

[4] Chen M., Xie Y., Chen H., Qiao Z., Qian Y. (2001). Preparation and characterization of metal sulfides in ethylendiamine under ambient condition through a γ-irradiation route. J. Colloid Interf. Sci..

[5] Khan S., Je M., Ton N.N.T., Lei W., Taniike T., Yanagida S., Ogawa D., Suzuki N., Terashima C., Fujishima A. (2021). C-doped ZnS-ZnO/Rh nanosheets as multijunctioned photocatalysts for effective H2 generation from pure water under solar simulating light. Appl. Catal. B Environ..

[6] Ali H.E., Nasef S.M., Gad Y.H. (2022). Remediation of Astrazon blue and Lerui acid brilliant blue dyes from waste solution using amphoteric supraparamagnetic nanocomposite hydrogen based on chitosan prepared by gamma rays. Carbohydr. Polym..

[7] (2012). Editorial, Site selective reactions: Nature chemistry. Nat. Chem..

[8] Brixner T., Pfeifer T., Gerber G., Wollenhaupt M., Baumert T., Hannaford P. (2005). Optimal control of atomic, molecular and electron dynamics with tailored femtosecond laser pulses. Femtosecond Laser Spectroscopy.

[9] Decker C. (1996). Photoinitiated cross-linking polymerization. Prog. Polymer. Sci..

[10] Peiffer R.W., Scranton A.B., Bowman C.N., Peiffer R.W. (1997). Photopolymerization: Fundamentals and Applications.

[11] Sullivan K.K., Boamah M.D., Shulenberger K.E., Chapman S., Atkinson K.E., Boyer M.C., Arumainayagam C.R. (2016). Low energy (<20 eV) and high-energy (1000 eV) electron- induced methanol radiolysis of astronomical interest. Mon. Not. R. Astron. Soc..

[12] Böhler E., Warneke J., Swiderek P. (2013). Control of chemical and synthesis by low energy electrons. Chem. Soc. Rev..

[13] Abdoul-Carime H., Bald I., Illenberger E., Kopyra J. (2018). Selective synthesis of ethylene and acetylene from dimethyl sulfide cold films controlled by slow electrons. J. Phys. Chem. C.

[14] Hla S.-W., Rieder K.-H. (2003). STM control of chemical reaction: Single molecule synthesis. Annu. Rev. Phys. Chem..

[15] Blackwell D.D., Chen F.F. (2001). Time resolved measurements of the electron energy distribution function in helicon plasma. Plasma Sources Sci. Technol..

[16] Szczerbinski J., Gyr L., Kaeslin J., Zenobi R. (2018). Plasmon driven photocatalysis leads to products known from E-beam and X-ray induced surface chemistry. Nano Lett..

[17] Bernardi M., Mustafa J., Neaton J.B., Louie S.G. (2015). Theory and computation of hot carriers generated by surface plasmon polartitons in noble metal. Nat. Comm..

[18] Illenberger E., Momigny J., Baümgartel H., Franck E.U., Grünbein W. (1992). Gaseous Molecular Ions: An Introduction to Elementary Processes Induced by Ionization.

[19] Fabrikant I.I., Eden S., Mason N.J., Fedor J. (2017). Recent progress in dissociative electron attachment: From diatomic to biomolecule. Adv. Atom. Mol. Opt. Phys..

[20] Abdoul-Carime H., Thiam G., Rabilloud F., Charlieux F., Kopyra J. (2022). Chemistry in acetonitrile-water films induced by slow (<15 eV) electrons; application to the Eath and space chemistry. ACS Earth Space Chem..

[21] NIST, Webbook of Chemistry. https://webbook.nist.gov/cgi/cbook.cgi?ID=107-15-3&Units=SI.

[22] Matsuura K., Muto H. (1993). Electronic structure of acetylene radical anion with a trans-bent form. J. Phys. Chem..

[23] Ha T.-K., Suter H.U., Nguyen M.T. (1996). Is acetylene radical anion with a tran-bent form observed in matrix experiment? An ab initio study. J. Chem. Phys..

[24] Chandrasekhar J., Kahn R.A., von Ragué Schleyer P. (1982). The preferred structure of C_2_H_2_^−^. Chem. Phys. Lett..

[25] Matejcik S., Kiendler A., Stamatovic A., Märk T.D. (1995). A crossed beam high resolution study of dissociative electron attachment to CCl4. Int. J. Mass. Spectrom. Ion Proc..

[26] Desfrançois C., Bouteiller Y., Schermann J.P., Radisic D., Stokes S.T., Bowen K.H., Hammer N.I., Compton R.N. (2004). Long-Range Electron Binding to Quadrupolar Molecules. Phys. Rev. Lett..

[27] Abdoul-Carime H., Mounier F., Charlieux F., André H. (2023). Correlated ion-ion/neutral time of flight mass spectrometer. Rev. Sci. Instrum..

[28] Denifl S., Ptasinska S., Probst M., Hrusak J., Scheier P., Märk T.D. (2004). Alectron attachment to the gas phase DNA base cytosine and thymine. J. Phys. Chem. A.

[29] Frisch M.J., Trucks G.W., Schlegel H.B., Scuseria G.E., Robb M.A., Cheeseman J.R., Scalmani G., Barone V., Petersson G.A., Nakatsuji H. (2016). Gaussian 16, Revision C.01.

[30] Chai J., Head-Gordon M. (2008). Systematic optimization of long-range corrected hybrid density functionals. J. Chem. Phys..

[31] Kendall R.A., Dunning T.H.J., Harrison R.J. (1992). Electron affinities of the first-row atoms revisited. Systematic basis sets and wave functions. J. Chem. Phys..

[32] Thiam G., Rabilloud F. (2021). Multi-basis-set (TD-)DFT methods for predicting electron attachment energies. J. Phys. Chem. Lett..

[33] Allouche A.-R., Gabedi A. (2011). User Interface for Computational Chemistry Softwares. J. Comput. Chem..

[34] Vinogradoff V., Duvernay F., Danger G., Theulé P., Borget F., Chiavassa T. (2013). Formaldehyde and methylamine reactivity in interstellar ices analogues as a source of molecular complexity at low temperature. Astron. Astrophys..

