# Supplementary material for: Experimental and Theoretical Investigations of the Fragmentation of Ethylenediamine Induced by Low-Energy (<10 eV) Electrons
Source: Molecules. 2023 Dec 28;29(1):191. doi: 10.3390/molecules29010191 (PMC10780159; doi:10.3390/molecules29010191)

Table S1: Energy (in eV) for the production of anion and corresponding neutral fragment(s), calculated at ωB97x/aug-cc-pvtz level of theory at 0K and Gibbs free energy at 298 K. The labels H(a), H(b) are shown in Figure 4a.

Reaction	Fragmentation Energy (0 K)	Gibbs Free Energy (300 K)
$EDA \rightarrow (EDA\text{-}H)^- + H$		
(a) N-H(a)	4.68	3.31
(b) N-H(b)	4.05	3.30
(c) C-H(a)	5.26	3.96
(d) C-H(b)	4.63	3.89
(e) $EDA \rightarrow NH^- + H + CH_2CH_2NH_2$	7.91	6.50
(f) $EDA \rightarrow NH^- + CH_3CH_2NH_2$	3.24	2.55
(g) $EDA \rightarrow CN^- + 2H_2 + CH_2 + NH_2$	8.61	5.94
(h) $EDA \rightarrow CN^- + 2H_2 + CH_2NH_2$	3.32	1.42
(i) $EDA \rightarrow CN^- + H_2 + H + CH_3NH_2$	3.74	1.93
$EDA \rightarrow C_2H_4^- + 2\ NH_2$	5.91	4.40
$EDA \rightarrow C_2H_4^- + N_2H_4$	2.72	2.04
$EDA \rightarrow C_2H_2^- + 2\ NH_3$	2.95	1.83
$EDA \rightarrow (C\text{-}NH)^- + H_2 + CH_3NH_2$	3.02	1.87
$EDA \rightarrow (C\text{-}NH)^- + H_2 + CH_2NH_2 + H$	7.26	5.43
$EDA \rightarrow (NC\text{-}H)^- + H_2 + CH_3NH_2$	3.15	1.70
$EDA \rightarrow (NC\text{-}H)^- + H_2 + CH_2NH_2 + H$	7.39	5.26

EDA → NH_2^- + $CH_2CH_2NH_2$	3.30	2.46

Figure S1 : NH^- (a) and CN^- (b) anion yield. The dashed lines are guide-to-the-eye.

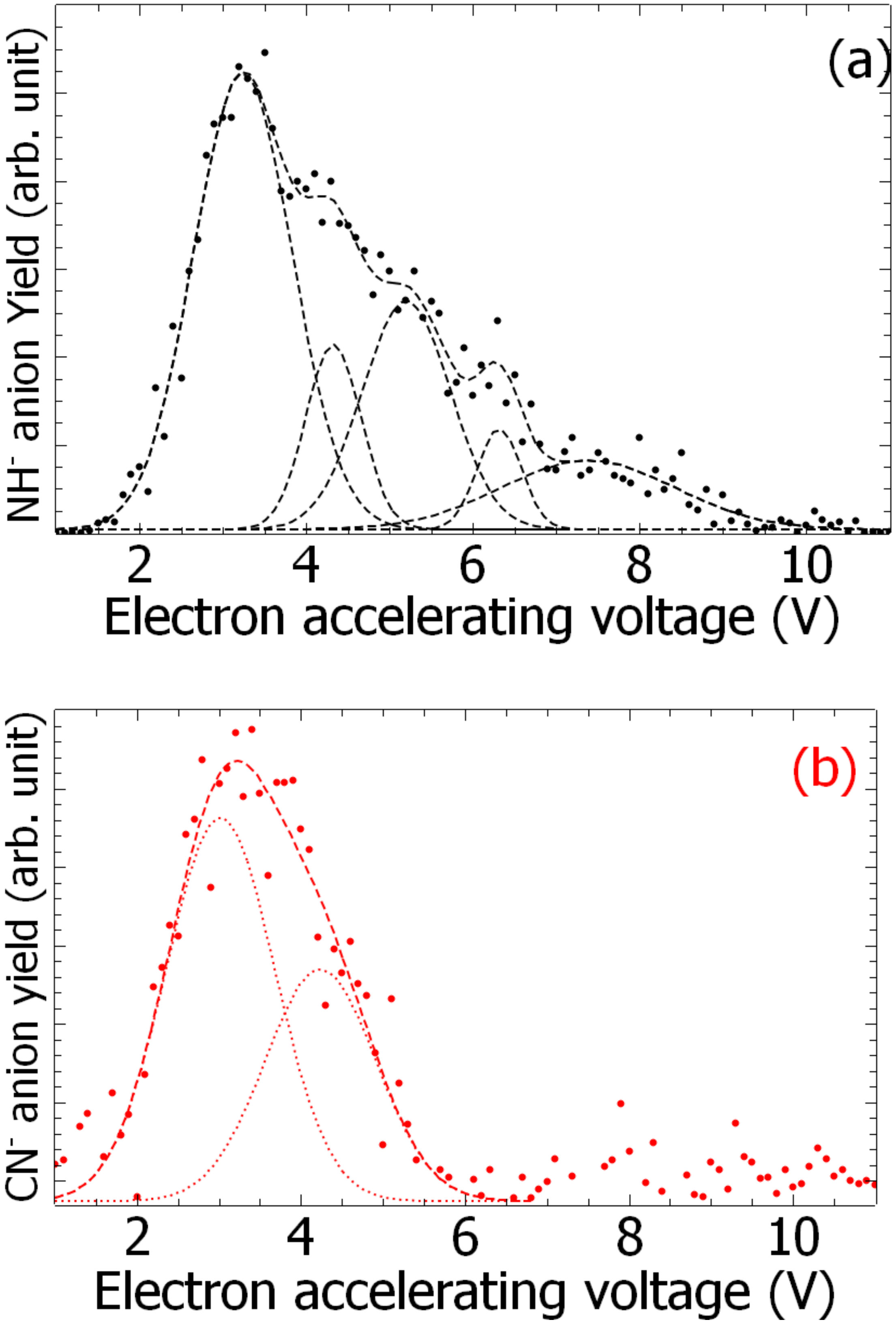

Supplement: Supplementary file 1 [file molecules-29-00191-s001.zip › molecules-2751503-supplementary.pdf]
